# Notes of a Few Cases of Cholera Treated in the Summer of 1849; Showing the Comparative Curative Powers of Sulphate of Quinine in Large Doses

**Published:** 1854-09

**Authors:** F. W. Sargent


J. *Notes of a few cases of Cholera treated in the Summer of* 1849; *showing the comparative curative powers of Sulphate of Quinine in large doses.* By F. W. SARGENT, M.D.

It would be impossible to select a disease concerning which there is more discrepancy of statement and opinion upon every point relating to it, and especially regarding its treatment, than *Cholera Asphyxia.* Probably, one fruitful source of this discord is to be found in the fact that different stages of the disease have been confounded. When A. states that he has been generally successful in the treatment of cholera by Calomel, and B. says that he has been as uniformly unfortunate, they probably had not reference to the same form, or stage, or degree of malignancy of the disease.

In the summer of 1849 several temporary hospitals were opened in this city for the reception of cholera patients. Dr. Wm. B. Wilson and myself were placed in charge of the one which was located on the north side of Cherry street, west of sixth street. We made accurate notes of all the cases of what we considered to be true *cholera asphyxia*, or *Asiatic cholera*, both of those which were brought to the hospital, and of those—much fewer in number—which we saw in private practice.

Dr. Wilson is since dead. Many of the notes now before me were taken by his own hand; and it gives me great pleasure, even at this late day, to bear willing, though mournful testimony to his many noble and endearing qualities. His zeal in his chosen profession; his excellent powers of observation; his soundness and accuracy of judgment; the amenity of his temper and manners; the simplicity, honesty and strength of his friendship, so unpretending, yet so reliable; these and many other admirable characteristics, both intellectual and moral, rendered his early death a great loss and a subject of deep regret to his friends, professional and lay.

> "The good die first, while they
> Whose hearts are dry as summer dust,
> Burn to the socket!"

The following pages furnish a brief summary of the treatment which we pursued in the collapse of cholera. We quote only a few of the cases which we recorded, as all would be unnecessary and uninteresting. For the same reason we say nothing concerning the simple diarrhœa, of which several cases were sent to the hospital, and which recovered under ordinary treatment. With reference to that form of diarrhœa which, from its history and symptoms and the great degree of prostration which it speedily induced, we regarded as being the initiatory stage of true cholera, we shall only remark that we found it to yield satisfactorily to small doses of calomel, opium and sugar of lead, or to the administration of the following solution, repeated every hour or two:

| | | |
|---|---|---|
| ℞. | Quiniæ sulphatis . . . . . | gr. i. |
| | Ferri sulphatis . . . . . | gr. ½ |
| | Acid. sulphuric. arom. . . | gtts. iij. |
| | Aquæ fluvialis . . . . . . | ℥j. |

To this we sometimes added a few drops of laudanum, or administered in addition laudanum by enema. We prescribed strict rest in bed and a farinaceous diet. We did not lose a single patient from either form of diarrhœa.

The number of patients whom we saw in the collapse of cholera, in the hospital and in private, was thirty-six, of whom twenty-one died.

We first employed calomel combined with camphor and opium, with sugar of lead and opium, &c.

Case 1. An Irish woman, æt. 21, brought from Bedford street, on the 2d of July, at 2½ o'clock, P. M. Her brother declares that she has had no diarrhœa; and the physician who was called to her certifies that since 8 o'clock P. M. of July 1st she has had no evacuation from her stomach or bowels, but cramps in her legs and feet and abdomen, and that her skin has been cold.

*Present condition*, 2½ *P.M., July 2d.* No pulsation of the artery at the wrist or elbow; femoral artery beating feebly at the groin; action of heart feeble, but quiet and regular, sounds faint but distinct; skin cold, shrunken, bluish, and covered with a cold moisture so copious as amply to compensate for the absence of the usual evacuations from the stomach and bowels; breath and tongue cool; voice faint, but neither stridulous nor husky; eyes sunken, injected and without expression; mind sluggish, but clear when aroused; severe and frequent cramp in muscles of legs and feet, fore-arms and hands, and also of abdomen.

The physician who first saw her had ordered her to take every hour 2 grs. of calomel and gr. j. of camphor; this was continued, and in addition we gave ℥ss. of beef broth with ʒj. of brandy every half hour; hot applications were made to various parts of the body, absolute rest was enforced, and ice was allowed freely.

The patient was dead in one hour.

CASE 2. An American, æt. 67, of temperate habits and very fair muscular development, has had diarrhœa exactly one week, with thin and, as he says, dark brown discharges; has had no nausea nor vomiting; about six hours ago he became sick at the stomach, began to feel cold, and had cramps in his legs, not extending above his calves, however.

Admitted at 7½ A.M., July 3d. *Present condition.* Skin bluish, slightly shrivelled; countenance sunken, face not cold; eyes injected and dryish; arms cold as high as the elbows; tongue quite cold, brownish, moist; voice husky and feeble; pulse just perceptible at the wrist; sounds of heart distinct, but feeble; intellect clear, but sluggish and indifferent to every thing, excepting to pain in the belly, which occasionally annoys him. He has had four thin, bluish looking evacuations from the bowels within an hour, each discharge being about ℥iv., and of a heavy, sickening odor.

Ordered to take, every half hour, calomel gr. j., camphor grs. ij., acetate of lead grs. ij.; in addition, ʒj. of laudanum was given by enema; ℥ss. of beef-broth with ʒj. of brandy every fifteen or twenty minutes; ice in small pieces as often as desired.

No good effect was produced, and the patient died at noon.

We recorded the history and progress of five other cases, treated after the same general plan. But we could not satisfy ourselves that we accomplished any thing by it, for, with one exception, all who were treated after this method died, no commencement of reaction having been manifested in the other six.

We tested in four cases "*Ayres' plan*" of treatment, giving gr. j. of calomel with gtt. j. of laudanum every five minutes. This method pleased us no more than the other; indeed, it was exceedingly disturbing to the patient, and very wearying to the attendants. Not one of the four recovered, nor did any one seem to be in the slightest degree benefited.

It is interesting to observe the great contrariety of opinion respecting the value of mercury in cholera, whether employed in the manner advised by Dr. Ayre or in any other, as expressed in the elaborate Reports on Cholera just published under the auspices of the Royal College of Physicians of London.

We made use of *Chloroform* in a few cases, as in the following:

CASE 3. A Fisherman, æt. 30, of rather intemperate habits, of powerful muscular development, and noted among his fellows for his determined character, was reported to us as having been attacked with cramps in the legs about four hours before his admission to the hospital, on the 10th of July. He had diarrhœa for three days previously, but sought no means of relief, excepting by the addition of "*a pinch of Cayenne pepper*" to his favorite glass of brandy, and by dispensing with his usual allowance of water with the spirit; his appetite had remained good, and he had pursued his customary labors, until to-day, although he had become considerably weakened by frequent alvine evacuations. Soon after he was seized with cramps he commenced vomiting, and the passages from his bowels became more frequent. The physician who first saw him, immediately after the occurrence of cramps, prescribed grs. ij. of calomel and 10 drops of laudanum, every half hour; stimulating frictions to his limbs and trunk, and, instead of water, a table-spoonful of ice-cooled "cayenne pepper-tea," as often as the patient felt thirsty. These directions were complied with until he was brought to the hospital, four hours after the invasion of cramps, at 11 o'clock, A.M.

*Present condition.* Skin of extremities cold to the trunk, body warm; fingers and toes shrivelled and bluish; tongue cooler than natural; voice clear, but feeble; mind clear and determined; muscular strength exceedingly reduced, so that with the strongest desire to rise from the bed to answer a call to stool, he was utterly unable to do so; cramps very severe and painful in muscles of limbs and abdomen, the muscles being contracted into very hard knots during the persistence of the spasm, though at other times the soft parts are inelastic and doughy to a great degree; pulse very feeble, 100 per minute, somewhat irregular; action of heart feeble, confused and languid, the sounds not always separable; had three very large evacuations from stomach and bowels in the course of the first half hour after his admission, the ejections being accomplished with great force, as from a large syringe, and consisting of the ordinary rice-water matter, very offensive to the smell when the nose was applied near to it.

*Ordered*,—Grs. ij. of camphor, dissolved in gtts. xl. of chloroform, to be given every half hour; ice to be allowed freely; heat to be applied to the feet and trunk; ℥ss. warm beef-broth every fifteen minutes.

The patient died between three and four o'clock in the afternoon. His evacuations became infrequent after the first hour from his entrance, and he was very quiet and comparatively free from pain and cramp. But after his death we found his stomach and bowels abundantly filled with matter similar to that which he had expelled during life, so that we could not infer that the medicine had exercised any influence in diminishing the amount produced. His quietude and freedom from suffering, as well as the cessation from the purgation and emesis, were rather due to increasing exhaustion and insensibility of his nervous system.

Case 4. Very similar to the last in all essential features, so that they could be very properly associated in comparison. The patient, a robust man, æt. 35, having had diarrhœa for four days, suffered from cramps and vomiting for three hours previous to his admission, on the morning of the 10th of July, having had no medical treatment whatever; he possessed an advantage over the last patient in being an honest member of a temperance society.

The treatment was precisely similar to that of the last case, excepting that 30 drops of oil of turpentine were substituted for the 2 grs. of camphor. The patient died in five hours after his admission, his evacuations having become small and infrequent, (but two during the last four hours,) and the cramps having ceased entirely after the first hour; the alimentary canal, however, was found after death pretty well filled with rice-water matter.

Case 5. R. K., æt. 45, white, an ostler by occupation, intemperate in habits, was admitted at 2½ o'clock, P.M., July 9th. He had diarrhœa for several days, which, however, had become so much more severe to-day that while engaged in bleeding a horse he had three evacuations, which he said were very abundant and looked like dish-water ; cramps and vomiting commenced at 12 o'clock, the matter from the stomach resembling that expelled from the bowels. A physician saw him at one o'clock, and ordered him grs. iv. of calomel with grs. ij. of opium, and sent him to the hospital.

*Condition at* 2½ *o'clock.* Skin cold and wet, breath and tongue cool ; fingers shrivelled ; nails not blue ; cramps in legs and arms, none in abdominal muscles ; muscles generally very soft, retaining impressions made in them like dough ; face and lips bluish, features shrunken and pinched ; the whole body so collapsed that Dr. Wilson, who knew him, thought he must have lost at least 30 lbs. weight within a few days. Intellect perfectly clear ; voice feeble, sometimes husky ; pulse frequent and very feeble, but easily counted ; sounds of heart confused, action rather violent and tumultuous ; restless ; frequent, prolonged and forcible inspirations ; complains of a sense of suffocation.

V. S. ad ℥xij. ; the blood flowed very sluggishly, requiring constant rubbing of the arm for its removal ; it was very thick and dark colored. Chloroform gtts. xl. with oil of turpentine gtts. xxx. were administered every half hour ; ℥j. of brandy with ʒj. of laudanum were given by injection ; ℥ss. of beef-broth was ordered every fifteen minutes ; ice was allowed freely, and hot applications were made to the feet. The patient had no cramps nor vomiting, nor purging after the bleeding ; the skin became warmer, first at the feet and gradually over the whole surface, and the face and lips assumed a natural color within an hour from the venesection. He died, however, at 5¼ o'clock, P. M., having become delirious an hour before death.

At the post-mortem examination, made 4½ hours after his demise, an abundance of the rice-water matter was found in the alimentary canal.

We may remark that we attached no importance to the fact that the skin became warmer after bleeding, as it was unaccompanied by corresponding improvement in the action of the heart, and in the state of the pulse. We had frequently noticed, what indeed almost every essay on cholera has commented upon, that the temperature of the body very frequently rises an hour or more before death in this disease, and perhaps more commonly soon after life has terminated.

We thus recorded accurately the histories of four patients whom we treated with chloroform, combined with camphor or oil of turpentine, laudanum being given p. r. n. by enema, and the diet and other matters of regimen being the same as already indicated. These four died, the medicine having been powerless to produce reaction, though the evacuations became less frequent. We did not administer chloroform or ether by inhalation.

We had read with much interest, in the volumes of the London Medical Gazette for 1847 and 1848, a paper by Dr. C. W. Bell. This gentleman had seen great numbers of cholera patients in India, and was struck with many points of similarity between this disease and intermittent fever. He found in the early stage of collapse that bleeding was generally curative, and also beneficial when resorted to a little later; and that a combination of quinine and iron was highly efficacious when collapse was fairly established. We resolved to give his opinions a trial, especially as the treatment by free bleeding had been found to be about as successful as any other, during the previous visitations of cholera in this city.

We had already resorted to bleeding in several cases of rather advanced collapse, after having first tried, in the same persons, one or other of the various plans to which we have alluded, and found that we were not likely to succeed. But we gained nothing under these circumstances, excepting, possibly, a slightly accelerated fatal termination of the lives and sufferings of our patients. In all these cases the blood was abstracted from the veins only by dint of constant rubbing of the arm, and then it escaped drop by drop, thick and dark-colored.

The writer, however, met with two cases in private practice, in which the stage of collapse seemed to be just forming, and in which he resorted to venesection with great advantage.

CASE 6. Ellen Murphy, an Irish girl, æt. 26, domestic in a private family, health said to have been uniformly good. On Saturday, 21st of July, she dined very heartily on corned-beef and cabbage; went to bed between 10 and 11 o'clock, feeling as well as usual; was awakened after midnight with severe pain in abdomen and diarrhœa. These symptoms continued during the following day, (Sunday,) and had become so much worse by evening that I was sent for.

*Eight and a half o'clock, P. M., July* 22*d*. Pulse feeble; heart beating vigorously and regularly, sounds distinct; skin warm and moist; face so much sunken as to have excited the alarm of the lady of the house, who had just returned to town, having passed the day in the country; eyes surrounded with a dark circle; tongue clean, moist and warm; voice feeble; strength much reduced; patient restless and apprehensive, complaining of oppression in breathing and of discomfort, and occasionally of pain at the pit of the stomach and the præcordial region; no cramps; has had six very large evacuations from bowels within the last three hours, consisting of a thin gruel-like matter, slightly roseate in color, exceedingly nauseous to the smell, and expelled with sudden violence; the stomach had been emptied almost as frequently as the bowels of a fluid resembling the discharges *per anum*, but less offensive in odor.

*Ordered*, gr. ½ calomel, grs. ij. acetate of lead and gr. ½ opium, to be given every half hour, in as little water as possible; a mustard plaster to be applied to the epigastrium, and others to the calves of the legs; ice freely; ʒj. laudanum by enema. At 10 o'clock I saw the patient again, and found that the powders had been rejected within a few minutes after they had been swallowed, and that every enema, though carefully administered in the quantity only of ℥ss., had been expelled almost so soon as the pressure made upon the anus was withdrawn. Surface, whereever exposed,—as the neck, upper part of chest, arms, hands and face—cold and moist, on legs and trunk warm; (the patient lay upon a feather bed and was covered with a blanket;) pulse decidedly more feeble and frequent than before; heart still beating vigorously, without confusion of sounds; tongue cold and moist; voice husky at times; patient more restless and oppressed than before, says she thinks her "lungs are stopped up;" frequent sighing and yawning; has had four evacuations from bowels since last visit, exclusive of the mere expulsion of the enemata; within the last half hour has had slight cramp twice in the legs.

V. S. ad ℥xvj.; the blood was not particularly dark in color and flowed freely, the respiration becoming relieved and the patient tranquil during its escape from the vein. In the course of a half hour the pulse became fuller, the skin uniformly warm, all restlessness ceased, and the uncomfortable sensations at the epigastric region disappeared.

Left provisional directions to administer an anodyne enema if the bowels should be moved, and the powders first prescribed in case of return of the vomiting.

*Twelve o'clock, midnight.* Skin rather warmer than natural all over the body and limbs; patient perfectly composed, has dozed from time to time, has no oppression, nor nausea, nor pain in abdomen; has had no evacuation, nor disposition to purging or emesis; no cramp since the bleeding; pulse 90, fuller and stronger than before.

*July 23d, 8 A.M.* Patient slept well during the night; had no evacuation from stomach or bowels; passed, early this morning, about ℥vj. of turbid-looking, darkish urine; she feels perfectly well, but languid and weak.

*Ordered* her to remain in bed all day; to take for nourishment farina prepared with milk, flavored with brandy; to take ice if she should be thirsty, and gr. j. of quinine with gr. j. sulphate of iron, in solution, every three hours.

*Four, P.M.* Has been well all day, and feels much stronger; bowels not moved.

*Directed* her to continue the medicine for a few days, and to be very careful of her diet, and to avoid fatigue as much as possible. Advised her also to take an injection if her bowels should not be moved spontaneously by morning. The girl had no further difficulty.

Case 7. July 25th, 6 P.M. Was called to see a young married lady, æt. 25, whose health was remarkably good, and whose physical development was better than that of most ladies of her class. She had diarrhœa for three days, which she had endeavored to arrest by means of chalk-mixture. She continued to attend diligently to her customary house duties, exposing herself to considerable fatigue, the weather being very warm, and had not taken the medicine with much regularity. This afternoon she had ridden a mile or more in an omnibus in the city, and had walked several squares.

*Present condition, 6 P.M.* Face very much collapsed; eyes sunken, with dark areolæ beneath; skin cool and moist; tongue cooler than natural to the touch; voice feeble, at times whispering, not hoarse; pulse feeble, and occasionally irregular; heart beating violently and now and then tumultuously; (the patient I knew to be free from disease of the heart!) sounds of heart distinct; has had severe cramps in the legs for an hour and a half, and in the abdomen during the last half hour; has had four large evacuations from bowels in the last two hours; (was compelled to stop at the house of a friend as she was returning home, to relieve her necessities!) Her discharges were described to me as being thin and bluish, and "awfully offensive, as if her insides were mortifying;" she has had neither nausea nor emesis; restless and sighing.

I immediately determined to bleed this patient, and while I was preparing to do so, she besought me to allow her to go into the next room to stool. I insisted upon her endeavoring to control the disposition, and lying quietly upon the bed as she was.

V. S. ad ℥xxij; the blood flowing at first sluggishly, and by dint of rubbing the arm, gradually the stream increased in size and in the length of the jet, the color brightened, and the pulse became fuller; the oppression and jactitation subsided entirely, the disposition to purgation ceased, and the patient expressed herself as completely relieved. In the course of a half hour the skin became warm over the body.

Encouraged by the progress of the case last reported, I gave merely provisional directions similar to those recommended in that instance—enjoining simply perfect repose in bed, the moderate use of ice if thirst should prove troublesome, and arrow-root prepared with milk for nourishment, to the amount of ℥iv. every hour, if it should seem to suit the stomach.

10 *o'clock, P. M.* Patient has been entirely undisturbed by any unpleasant sensation or symptoms; bowels not moved, passed about half a pint of dark-colored urine at 9 o'clock; has slept several times.

*July 26th*, 8 *A. M.* Passed the night very comfortably, having slept as much as usual, she thinks; bowels not moved, urine passed naturally, and but slightly darker than common; appetite good; feels somewhat dizzy and weak when she sits up in bed, otherwise well.

*Ordered* ℞. Quiniæ sulphat.; ferri sulphat. aa. gr. j.; aquæ fluvial, ʒij., acid. sulphuric. aromat. q. s. ut ft. solutio, to be repeated four times daily, for a few days; also directed the patient to be careful in her diet, and in the amount and kind of exercise she should take. There was no further trouble in this case.

Thus it will be seen that, in both these instances, the bleeding seemed at once to arrest the morbid actions which had taken place, and to produce a state of constipation, so far as the abnormal excretions were involved.

We were disappointed in our experiment with quinine and sulphate of iron, used in the way recommended by Dr. Bell, in cases in which collapse could fairly be said to exist. We recorded the histories of three such examples, of which the following may serve as specimens.

Case 8.—A black girl, æt. 19, according to her own account, but looking as if she were 24 or 25 years old, was attacked last night at 9 o'clock, with diarrhœa and vomiting, and soon after with cramps in the legs. Had been free from any sickness until evening; had been washing clothes during the day; thinks she brought on her attack by eating too many strawberries. Her bowels were moved six or eight times during the night, and she had vomited, perhaps, as often. Had no medical advice until 10 o'clock this morning, when the physician who saw her ordered her to take gr. j. of calomel, grs. ij. of camphor, gr. j. of acetate of lead, and gr. ½ of opium, every hour, which she has regularly complied with. (The physician sent a copy of the prescription.)

*Admitted at half past four o'clock, July 3d.*—skin cold wherever exposed to the air, warm where covered; face moderately collapsed; eyes decidedly, though not excessively sunken; lips and tongue slightly bluish and cool; fingers shrivelled, nails bluish; pulse exceedingly feeble; heart beating feebly, sounds clear and distinct; respiration somewhat labored, 20 per minute; abdomen full, not distended; vomited, soon after her admission, about ℥ij. of a light brown fluid; no cramps since morning; no purging since noon; her friends who accompanied her, say that she seems much worse than at 12 o'clock.

*Ordered.*—The solution of quinine and iron, to be given every half hour, (sulph. quinine, grs. xx; sulph. iron, grs. ix; elixir vitriol, ♏. xl; aquæ Oiss., dose, ℥ij.;) the surface to be rubbed with an alcoholic solution of salt; hot applications to be made to the feet and trunk; ice to be allowed freely; warm beef-broth, ℥ss. every fifteen minutes.

The quinine solution seemed to relieve the nausea immediately, so that she had no farther evacuation from the stomach or bowels; but no other improvement took place in her condition, and she died at half past seven o'clock.

Case 9.—A black man, æt. 35, native of South America, of very large muscular development, and intemperate habits, says he was attacked on the 1st of July, with great weakness and vomiting, but no diarrhœa; the fluid from the stomach, he says, was of a whitish color.

*Admitted at* 7½, *A. M., July 3d.*—Skin cool on face, neck and arms, cold below the knees, trunk warm; features not much shrunken, eyes somewhat sunken; intellect perfectly clear and collected; tongue warm, whitish in the centre, red at edges; pulse feeble; heart's action feeble, but regular, both sounds distinct; cramp in legs.

*Ordered* the quinine and iron solution, as before, every half hour; stimulating frictions of surface; hot applications to feet; ice freely enough to relieve his thirst; warm broth.

The patient died at midnight. He had no evacuation from his bowels until 11 o'clock, A. M.; between which time and 4, P. M., he had four passages of a thin, dark-brown fluid, containing small dark grains, looking like sage, and numerous whitish flocculi, resembling finely cut vermicelli; each evacuation was about ℥iv. in quantity. He had no cramps, nor emesis; complained of no pain, nor other discomfort, excepting of heat; though his surface became more and more reduced in temperature. We found that for a time, frictions of the surface with ice gave great relief to this annoying sensation of heat; but after a time the rubbing became rather disagreeable, and he begged not to be further annoyed by "any cussed rubbin"; for two hours before his death, no pulsation could be felt in the brachial or femoral arteries, and but very faintly in the carotids; at 4 o'clock, passed from urethra ℥ss. of a thick whitish fluid, having a urinous odor, and the same quantity again at 5 o'clock; an hour before death he became delirious. The warm broth was very disagreeable to him, increasing the sensation of heat of which he complained, so that we soon abandoned it, giving him, instead, arrowroot prepared with milk, of which he took with cheerfulness, ℥j. every half hour, with ʒj. of brandy.

Having had some experience of the efficacy of sulphate of quinine given in doses of ten to twenty grains, in the treatment of pernicious intermittent fever; and surmising that, if Cholera were a form of this disease, the same remedy should be serviceable, and that if there were no relationship between them, the stimulating effects of the quinine upon the nervous system, would still render it advisable to resort to it, we determined to test the idea. The first instance in which we employed it, we recorded as follows:

CASE 10.—An Irishman, æt. 46, a laborer by occupation, intemperate, was admitted at 9½, A. M., July 13th. During the preceding night, he had had slight pain in his bowels, and four large watery evacuations; having been previously, as he assured us, perfectly well; went to work as usual at 6 o'clock, this morning, but was soon seized with violent cramps, frequent purging and vomiting, and was sent to the Hospital.

*Present condition*, 9½, *A. M.* Face pinched, eyes sunken, lips blue, temperature of tongue lower than natural; neck, chest and extremities cold; fingers shrivelled as if they had been macerating in warm water, nails purple; pulse just perceptible at the wrist; heart beating feebly and irregularly; respiration not much disturbed; violent cramp in left leg below the knee; very urgent for cold water.

10 o'clock.—He was put to bed, frictions of mustard and cayenne-pepper were made to his limbs, and bags of hot oats were placed about his body; he was allowed ice freely; ℥j. of beef broth was administered every half hour, and grs. xviij. of quinine, with gtts. xxx. of laudanum, and gtts. xx. of elixir of vitriol were given in a wineglassful of water.

11 o'clock.—Has had no cramp during the last half hour, and no evacuation from the stomach or bowels, since he took the quinine; skin warm, pulse becoming fuller and stronger, action of the heart perfectly regular; not at all restless; thirst very much abated; no headache, nor noise in the ears, nor disturbance of vision. Ordered grs. v. quinine in acidulated water, without laudanum.

1 o'clock, P. M.—Has just passed ℥ij. dark colored, turbid urine, coagulable by heat; condition very favorable.

3 o'clock, P. M.—Has been sleeping quietly, rather more than an hour; skin moist and warm; pulse 90. When he awoke, he asked for something to eat, and took ℥viij. of beef-broth; still thirsty, and is allowed to take cold water pretty freely.

7 o'clock, P. M.—Has slept the greater part of the afternoon; on awaking just now, passed nearly half a pint of urine, lighter in color than the last, and very slightly coagulable by heat.

*July 14th,* 8, A. M.—Had a very comfortable night, feels perfectly well, "barrin' the wakeness;" had a large, dark-colored, semi-solid evacuation from the bowels, accompanied with much wind, but no pain; his whole condition and aspect very much changed for the better; has a good appetite, which he is permitted to indulge on tea and bread and butter for breakfast and supper, and soup with rice for dinner.

Directed to take gr. j. each of quinine and sulphate of iron, four times daily.

Was discharged well, on the 16th of July, having had no relapse or other unpleasant symptom.

The following patient was seen by Dr. Carson and several other gentlemen, who happened to be in the ward at the time of his admission.

Case 11.—The cook and steward of a West India vessel, was admitted at 1½, P. M., July 17th. His habits are good, and his health always robust. He left Baltimore in the cars at 9, A. M. in excellent health, having had a natural stool at his usual time in the morning; towards the close of the ride from Baltimore to Havre-de-Grace, he became languid, drowsy and chilly; on getting into the boat at the latter place, he drank two large glasses of iced water in succession, and very soon experienced a sensation of pain and great oppression at the the pit of the stomach; cramps and vomiting soon commenced, accompanied later by purging; he was placed in a carriage immediately on his arrival at the depot in this city, and was brought to the Hospital; he had a copious evacuation of a watery fluid from his stomach and bowels, during the ride of half a mile.

*Present condition,* 2½ *o'clock, P. M.*—Surface generally cool; tongue clean, moist and cold, but not extremely so; skin wet with perspiration; pulse *very* feeble; fingers puckered like those of a washer-woman; voice feeble and husky; extreme thirst; restless to a great degree; respiration sighing; complains of feeling extremely hot, and oppressed in breathing.

*Ordered*, grs. x. quinine, and gtts. x. laudanum, in ℥ss. brandy; hot oats, in bags, were applied about his body and against his feet.

In the course of half an hour, his skin had become pleasantly warm, his restlessness and thirst had abated, his respiration had become regular without sighing, and his pulse was of a very encouraging force and volume; no vomiting, purging nor cramp after the first fifteen minutes from his admission; (just after his entrance, he ejected an abundance of the "rice-water" matter from his stomach and bowels, but there was no repetition of the discharges after he took the quinine.)

3½, P. M. The quinine, brandy and laudanum has just been repeated in the same proportions, there seeming to be a disposition to flag again; reaction followed as at first, and continued until about 5½, P. M., when it was thought advisable to administer a third dose, inasmuch as the patient seemed a little restless, and his pulse not quite so strong as before. After this his condition improved, and he went on steadily gaining. In the early part of the evening, he passed ℥iv. of dark reddish-brown urine. During the following days, he took sulphate of quinine and sulphate of iron, gr. j. of each, four times daily, until the 21st of July, when he was discharged cured.

Case 12.—James Owens, æt. 49, pretty well known as superintendant of the "State House," was admitted July 26th, at 9½, A. M. He had diarrhœa for a week; but on the night of the 25th, was seized with cramp in abdomen, hands and feet, accompanied with vomiting and increased purgation.

*Present condition.* Countenance sunken; eyes hollow; nose pinched and blue; lips purplish; tongue cool; skin of the hands shrivelled, moist and cold; nails blue; pulse very small, and very weak and irregular; action of heart feeble, and somewhat irregular, sounds distinct; muscles flabby and doughy; extremities cold, neck and face cold, trunk warm. While he was being undressed, he vomited about ℥iv. of a thin, bluish fluid, containing numerous flocculi of whitish matter in small fragments.

He was put to bed, hot oats in bags placed about him, and grs. xviij. of sulphate of quinine, with gtts. x. of laudanum in ℥ss. of brandy were administered; a piece of flannel wet with spirits of turpentine was laid upon the epigastrium; ice was freely allowed.

In the course of twenty minutes his pulse had become much fuller and perfectly regular; his lips grew slightly ruddy. At the end of an hour, the quinine, laudanum and brandy were repeated, reaction seeming to be at a stand-still. An hour and a half subsequently, (at about 12 o'clock,) a perfect reaction was produced, and he continued to recover. After the first dose of quinine, he had no return of vomiting or purging, and but one attack of cramp in one of his legs. At 7½ o'clock, he passed ℥viij. of darkish, muddy-looking urine; at 9 o'clock he fell into a sound healthful sleep.

During the two or three days following, he took gr. j. each of sulph. quinine and sulph. iron, four times daily.

But quinine, when administered in this way, did not answer our hopes in every instance. We employed it in sixteen cases, in the hospital and in private practice, and four of our patients died. The following notes may interest some of our readers.

Case 13.—Henry Smith, æt. 64, an Englishman, hale and hearty, of good habits, living at the "Preston Retreat" building. On the 9th of August he ate heartily of corned-beef and cabbage for dinner; in the afternoon, diarrhœa commenced and continued through the night, accompanied by cramps in his legs; to the latter he did not attach any special importance, as he was rather subject to them at night; the evacuations from his bowels were very large and thin, leaving no stain upon the paper which he used; he had no vomiting during the night, but frequent cramps at the pit of the stomach.

*Condition at 9½, A. M., August 10th.*—Skin blue and cold; nails blue, fingers very much shrivelled; muscles doughy; tongue and lips bluish and cool; pulse very feeble, varying from 60 to 90 beats per minute; heart correspondingly feeble and irregular in its action, both sounds distinct; voice feeble and husky; violent cramp in thighs, legs, feet and abdomen, none in arms; no nausea or vomiting; had a large evacuation at 9 o'clock, which was not examined, the man having persisted in going out to the privy in the garden.

*Ordered.*—Quiniæ sulph. grs. xv. in water; ʒj. of laudanum in ℥ss. of starch-water by enema; hot applications made to various parts of the surface; ice freely; ℥ss. beef-tea every fifteen minutes.

*At* 10 *o'clock*—He took an additional dose of grs. viiss. of quinine in ℥iss. water.

12½ *P. M.*—Pulse regular, 80 per minute, and of good volume and force; action of heart more vigorous, skin warm; tongue warm and less blue; breath warm; cramps much less frequent and severe; no evacuation since the first dose. *Ordered* grs. x. of quinine and gtts. x. of laudanum.

3½ *P. M.*—Condition still improved, no evacuation from stomach or bowels; no disturbance of senses, no noise in ears, no headache. Ordered grs. ij. of quinine, every hour.

6½ *P. M.*—Skin cold and wet, much less force of circulation than at last note, and less coloration of lips and tongue; at 5 o'clcck, he passed about ℥vi. darkish colored urine, and half an hour after, he insisted that he was strong enough to go into the yard to have his bowels moved, which he did; at 6 o'clock, he had another very copious evacuation from the bowels, looking, as his wife expressed it, like flakes of curdled milk floating in whey; he was permitted to cross the room and use a close stool, not feeling able to go out as before; since these evacuations, he has been sinking. He continued to fail from this time, and died at 1½, A. M. 11th August.

Case 14.—Esther Lawrence, æt. 42, chief female nurse at this Hospital, in person rather fat, but of a sound vigorous constitution. Has had diarrhœa a week; on the 25th July, she had an attack of cholera-morbus, apparently from eating too largely of tomatoes; on the 26th, she went over to the Blockley Hospital, where she had formerly resided as nurse, and spent the day in visiting her friends and the cholera wards; in the evening she again ate heartily of tomatoes; on the 27th, she attended as usual to her hospital duties, her diarrhœa still continuing; during the night she was almost incessantly vomiting and purging, and had cramps in her legs; at 8 o'clock, A. M. 28th July, she first consulted one of us, assuring us that she had a slight diarrhœa, but no vomiting, no cramp, nor any other unpleasant symptom; her pulse, the temperature of the surface, her strength, &c., indicated nothing contrary to her statements, and she was merely directed to keep in bed, and to take gr. ½ of calomel, gr. ¼ of opium and gr. j. of acetate of lead, every two hours; the diet was to consist exclusively of Arrow-root prepared with milk, and she was allowed ice.

Later in the day, we ascertained what the real history of her case had been, as above given.

9 *o'clock.*—Face very much altered, pinched and sunken; extremities cool and wet; heart beating strongly, sounds distinct; pulse feeble, 100 per minute; has had seven large watery evacuations since 8 o'clock, looking like soup containing very finely cut vermicelli; has had repeated attacks of cramp, and two evacuations form the stomach, the fluid ejected resembling very much that voided per anum; great restlessness and sense of oppression; mind depressed.

Ordered sulph. quinine, grs. xv.; elixir vitriol, gtts. x.; water, ℥ij., and laudanum, gtts. xx.: ice to relieve the thirst; hot irons to feet, and ʒj. of laudanum, ℥ss. brandy and ℥ss. starch water were administered by enema.

The medicine seemed to have no effect whatever upon her, the first dose was rejected from the stomach in half an hour after it was swallowed; it was repeated in a smaller quantity of water, but this was thrown up also; ℈j. of quinine mixed with powdered sugar, was applied to the surface at the epigastrium, the cuticle having been removed by the action of aqua ammoniæ; quinine was likewise administered by enema; chloroform was tried; but all was of no effect: the patient became more and more collapsed and died at 11 o'clock, P. M.

Case 14.—An Irishman, æt. 38, intemperate in habits, seemingly strong and healthy; he got drunk on the 6th of August, and continued so until the 9th, when he was seized with diarrhœa, accompanied by vomiting, severe cramps and great depression. From 8 o'clock, A. M. until 1½, P. M., he received no medical aid; he was then brought to the Hospital.

1½, *P. M.*—Complexion exceedingly bluish; eyes much sunken; tongue cold, clean and moist; breath cold: respiration difficult: voice extremely feeble: pulse scarcely perceptible at the wrist: heart pulsating feebly, both sounds clearly distinguishable: muscles doughy: while he was being conveyed from his home to the hospital, he vomited three times, and again while his clothes were being removed, preparatory to his being put to bed: the matter ejected was fluid, looking like dirty water, with whitish flocculi floating in it, and having a nauseous smell: very frequent and severe cramps in muscles of legs, arms and abdomen: thirst very great: restlessness extreme.

*Ordered* grs. xx. of quinine, gtts. xx. of elixir vitriol, and gtt. x. of laudanum, given without water: bags of hot oats were placed in contact with the surface: ice was allowed freely: warm beef-broth ℥ij. to be given every half hour.

As no improvement had taken place in a half hour, the quinine was repeated as above. Still no improvement occurred. A large vein was now opened at the bend of the left elbow, and a saline solution was thrown in as carefully and gently as possible, an operation which we found very difficult of execution, in consequence of the great restlessness of the patient. The formula which we employed, was as follows:

| | |
|---|---|
| Chloride of sodium, | ʒss. |
| Carbonate of soda, | grs. x. |
| Carbonate of potassa, | grs. x. |
| Sulphate of soda, | grs. v. |
| Phosphate of lime, | grs. v. |
| Water, | Oj. |

The solution was maintained at the temperature of the blood, and injected very slowly. We could introduce only six ounces. For a half hour, the patient seemed improved; during this time, we again repeated the quinine as at first given, but he speedily sank, and died at 3½ o'clock, P. M.

We have not thought it worth while to narrate the histories of all the cases of collapse, which we treated by quinine; their similarity would render our story too monotonous. They were sixteen in number, of which four died. Though too few to permit us to speak of them exeepting with reserve, they are yet sufficiently numerous, and contrast so favorably in their termination with the cases in which other methods of treatment were employed by us, as to warrant us in recommending our readers to make trial of this plan. Since the summer of 1849, the reporter has been so fortunate, or unfortunate, as not to have treated a case of malignant cholera, excepting in the solitary instance of one of the pupils at the Girard College, a boy of seven or eight years of age. In this case, the stage of collapse, though decidedly formed, was not far advanced, and five grains of quinine, repeated every hour until fifteen grains had been taken, brought about a complete and permanent reaction, without the aid of brandy or opium in any form.

We have purposely avoided all inquiry into the pathology of Cholera, our object being simply to present a few facts which came under our own observation, regarding the comparative efficacy of different methods of treating the disease.

To recapitulate, we have cited in all thirty-seven cases of cholera in the stage of collapse, including the one just alluded to: of these seven were treated with calomel in combination with sugar of lead, opium, &c., and of these only one recovered; four were treated according to "Ayres' plan," none recovered; four by chloroform, with camphor or oil of turpentine, none recovered; three by quinine and sulphate of iron, in small doses, without a single cure; seventeen by quinine in large doses, of which thirteen recovered; and two by bleeding, of which both recovered.

We scarcely venture to lay claim to originating this mode of employing quinine, although we have not at any time seen or heard of it elsewhere; it is hardly supposable, however, that some of our brethren of the South and West, where this medicine constitutes one of the great "staples" of consumption, should not have thus made use of it. "*Palmam qui meruit ferat.*"

